# Optimization of Pressurized Liquid Extraction of Lycopodiaceae Alkaloids Obtained from Two *Lycopodium* Species

**DOI:** 10.3390/molecules26061626

**Published:** 2021-03-15

**Authors:** Aleksandra Dymek, Jarosław Widelski, Krzysztof Kamil Wojtanowski, Paulina Płoszaj, Rostyslav Zhuravchak, Tomasz Mroczek

**Affiliations:** 1 Independent Laboratory of Chemistry of Natural Products, Medical University of Lublin, 1 Chodzki St., 20-093 Lublin, Poland; ensu@o2.pl (P.P.); tmroczek@pharmacognosy.org (T.M.); 2 Department of Pharmacognosy, Medical University of Lublin, 1 Chodzki St., 20-093 Lublin, Poland; jwidelski@pharmacognosy.org (J.W.); krzysztofkamilw@gmail.com (K.K.W.); 3 Rivnenskyi Nature Reserve, Natural Boundary “Rozvylka”, 34500 Sarny, Rivne Region, Ukraine; zhuravchak.ro@gmail.com

**Keywords:** *Lycopodium clavatum* L., *Lycopodium annotinum* L., *Lycopodium* alkaloids, pressurized liquid extraction, solid-phase extraction, HPLC/ESI-QTOF–MS

## Abstract

Alkaloids of the Lycopodiaceae family are of great interest to researchers due to their numerous properties and wide applications in medicine. They play a very important role mainly due to their potent antioxidant, antidepressant effects and a reversible ability to inhibit acetylcholinesterase (AChE) enzyme activity. This property is of immense importance due to the growing problem of an increasing number of patients with neurodegenerative diseases in developed countries and a lack of effective and efficient treatment for them. Numerous studies have shown that Lycopodiaceae alkaloids are a rich source of AChE inhibitors. In the obtaining of new therapeutic phytochemicals from plant material, the extraction process and its efficiency is crucial. Therefore, the aim of this work was to optimize the conditions of modern PLE to obtain bioactive alkaloids from two *Lycopodium* species: *L. clavatum* L. and *L. annotinum* L. Five different solvents of different polarity were used for prepared plant extracts in order to compare the alkaloid content in and thereby effectiveness of the entire extraction. PLE parameters were used based on multiple studies conducted that gave the highest alkaloids recovery. Crude extracts were purified using solid-phase extraction (SPE) on Oasis HLB cartridge and examined by HPLC/ESI-QTOF–MS of the highly abundant alkaloids. To the best of our knowledge, this is the first time such high recoveries have been obtained for known Lycopodiaceae alkaloids. The best extraction results of alkaloid-lycopodine were detected in the dichloromethane extract from *L. clavatum*, where the yield exceeded 45%. The high recovery of annotinine above 40% presented in *L. annotinum* was noticed in dichloromethane and ethyl acetate extracts. Moreover, chromatograms were obtained with all isolated alkaloids and the best separation and quality of the bands in methanolic extracts. Interestingly, no alkaloid amounts were detected in cyclohexane extracts belonging to the non-polar solvent. These results could be helpful for understanding and optimizing the best conditions for isolating potent AChE inhibitors.

## 1. Introduction

Species belonging to the Lycopodiaceae family, such as *Huperzia* or *Lycopodium,* are widely known around the world and have become the subject of numerous research. Due to the presence of alkaloids and their high biological activity, they have various properties and many applications in medicine [1]. A great number of plants such as *Huperzia serrata* (Qian Ceng Ta) have been used in Chinese folk medicine to treat many disorders, e.g., contusions, swellings, fever and blood disorders [2,3,4]. Currently, *Lycopodium* alkaloids are used in the treatment of aneurysms, chronic lung and bronchial diseases. They reduce inflammation of the digestive system, soothe the skin, reducing its irritation and itching [2,3]. The aerial parts of plants, especially *Lycopodium clavatum* are used as laxatives and diuretics, while they are commonly utilized as an aphrodisiac in South America [4]. There are also reports of the use of some *Lycopodium* genus in Southeast Asia and in the USA as an analgesic remedy to relieve rheumatic and muscle pains [5]. Increasing number in research studies indicate their application in homeopathy. *Lycopodium* extracts, used in alternative medicine, have shown hepatoprotective and anticancer effects for liver cancer, mentioned in many studies on animal models (including rats and mice) [6,7,8]. Furthermore, most in vivo and in vitro studies have confirmed that *Lycopodium* alkaloids have beneficial effects in the treatment of brain diseases. The inhibition of AChE activity was found in the rat cortex and hippocampus incubated in vitro with extracts of two *Lycopodium* species. Moreover, a significant decrease in AChE activity was also observed in vivo in these memory brain structures after administration of *Lycopodium* extracts to adult mice. This study provides the grounds for concluding that these alkaloids may exhibit positive effects on learning and memory and improves cognitive deficits [4,9]. Many scientific studies describe results proving alkaloid huperzine A (HupA) to be a great acetylcholinesterase (AChE) inhibitor. This alkaloid was obtained for the first time from Chinese medicinal herb *Huperzia serrata* belonging to the Lycopodiaceae family [4,5,6,7]. The HupA has a strong antioxidant activity against free radicals and β-amyloid responsible for cellular toxicity. Furthermore, it has the ability to reversibly and selectively inhibit the activity of AChE. This property gave hope in the treatment of different types of dementia such as Alzheimer’s disease, which is the fourth most common cause of death in developed countries. Cholinesterase inhibitors are the most commonly used first line pharmacotherapy effective in treating mild to moderate patients suffering from Alzheimer’s disease. Furthermore, some potent AChE inhibitors are derived from a natural source and most of them belong to the alkaloids, including physostigmine and galanthamine, isolated for the first time from plant materials [3,4]. Currently, a well-known alkaloid, galanthamine has a huge influence in the treatment of neurodegenerative disorders such as Alzheimer’s disease. However, the therapy consists only in increasing the concentration of acetylcholine (ACh) at the synaptic site in the brain, stopping the symptoms of the disease, improving memory and alleviating behavioral disturbances [4,10].

Moreover, the available drugs such as rivastigmine, galanthamine or donepezil are extremely limited and have some significant side effects. Many in vivo studies confirmed better pharmacokinetic properties of HupA. Unlike these drugs, HupA has better penetration through the blood–brain barrier and longer duration of AChE inhibitory action [11,12,13]. In addition to these unique activities, other pharmacological properties have been confirmed, including anti-inflammatory, antioxidant, neuroprotective, analgesic and antiepileptic activities [4,11,14,15,16]. These multiple actions may have breakthrough significance in the treatment of neurodegenerative diseases. Unfortunately, the commonly known *Huperzia serrata* is an endangered species. To this end, more research has focused on replacing plant resources with chemical synthesis to obtain this alkaloid and its analogs. However, this method is very expensive and not feasible for large-scale production [4,11]. To solve these problems and meet the current demand, new AChE inhibitors and the best methods for their isolation are still being sought. So far, there have been few reports on the chemical constituents of *Lycopodium clavatum* or *Lycopodium annotinum*, and there is almost no research on modern methods for their isolation [1,4,12,14]. However, the experience accumulated over centuries, and the widespread use of alkaloids inspires the search for new drugs in modern times. It has led to the identification of over 200 alkaloids from 54 *Lycopodium* species since the 19th century [6,17,18,19]. Numerous studies have allowed research to classify *Lycopodium* alkaloids into four groups, depending on the structural features, including: lycopodine, lycodine, fawcettimine and miscellaneous. Among them, for instance lycopodine, lycodoline, annotine, α-obscurine and many others have been tested towards the above-mentioned activities [4,5,9,15,20,21,22].

However, despite such high activity of these compounds, there is not enough data of their extraction and isolation methods. Until now, scientists have only used conventional methods to obtain bioactive *Lycopodium* alkaloids. No attempt has been made to use modern techniques and approaches to obtain potent strong AChE inhibitors. Standard methods for example liquid–liquid extraction or silica gel column chromatography were frequently used. However, scientists search for a factor that could increase the effectiveness of the extraction. Research have published several works using different organic solvents such as hexane, methanol, ethanol and many others in a different concentration. The use of these solvents has led to the isolation alkaloids and confirmation of many their properties like anticholinesterase, anti-inflammatory, antibacterial, antifungal and antiviral activities [5,23]. Konrath and coinvestigators [4] carried out a Soxhlet extraction and maceration with n-hexane as a solvent. The dried and ground aerial parts from two *Lycopodium* species: *Lycopodium thyoides* (Humb. and Bonpl. ex Willd.) and *Lycopodium clavatum* L. were extracted. The yield of these two extracts reached no more than 0.4% and allowed research to obtain the main alkaloids: lycopodine, lycodine or α-obscurine and to test towards anti-AChE activity [4]. In turn, Orhan and co-conductors [5] achieved higher extraction efficiency. They tested four extracts using maceration with four organic solvents and the alkaloid fraction from the aerial parts of *Lycopodium clavatum* L. In vivo studies confirmed that only the chloroform extract with the alkaloid fraction displayed the marked anti-inflammatory effect [5]. However, these methods required a huge amount of organic solvents and more time-consuming steps and generated a large amount of waste.

Da Silva and coinvestigators [1] used supercritical fluid extraction (SFE) to obtain the lycopodine from the *Lycopodium clavatum* L. The optimization of the extraction conditions was possible due to the influence of parameters such as temperature, pressure and time of pretreatment of the plant material. As a result of many studies, Da Silva has concluded that an increase in temperature and pressure are significant and gave a satisfactory yield of lycopodine above 20%. This methodology has been widely described in the chemical literature as a way to optimize many processes. What is more, it helps to evaluate the impact of several variables, thus reducing time and costs. Many scientists have tried to combine several techniques for better separating and purifying the extracts. For this purpose Zhang and co-conductors [23] used combination of some methods–ultrasonic assistant method, macroporous resin column chromatography and liquid–liquid extraction to separate and purify alkaloids HupA and huperzine B (HupB) from *Huperzia serrata* (Thunb.) Trevis. The optimization and special combination these methods finally led to the purification of the extracts and obtaining high values of these alkaloids [1,23,24,25].

The major disadvantages observed in the above studies were extract yield, extraction time, quantities and a kind of solvents. Pressurized liquid extraction (PLE), so-called accelerated solvent extraction (ASE) has become a promising approach to solve these problems. PLE is a novel extraction performed at elevated temperatures between 50 and 200 °C and high pressure 10–15 MPa with the use of the most common solvents (methanol, acetone, ethyl acetate, hexane, toluene and dichloromethane) that is similar to SFE. These parameters increase the extraction efficiency because they reduce the viscosity of the solvent, thus allowing better penetration of solvent molecules into the sample matrix. The flexibility to set numerous parameters like pressure, temperature, time or number of cycles during extraction, rinsing length allows scientists to obtain high-quality extracts. Therefore, in a relatively short time, this method allows one to optimize the best extraction conditions. Currently, this technique is the fastest, more economical and efficient extraction process with high extraction yield. Besides it is an easy, the repeatable process uses several factors to increase extraction efficiency [26,27,28,29].

This technique has been successfully used to extract various compounds, including alkaloids, in combination with different techniques coupled with chromatographic and spectrometric methods [30]. However, this is the first time that PLE with different solvents of different polarity has been utilized to obtain alkaloids from shoots of the two *Lycopodium* species. So far, there have been no studies using an innovative approach that would achieve a high yield of Lycopodiaceae alkaloids. Furthermore, no studies have been carried out to check an influence of different types of solvents on the obtained amount of potential AChE inhibitors. Owing to numerous limitations of conventional methods, the studies have been carried out to optimize PLE in the hope of finding a new, less time-consuming technique with reduced solvent consumption and increased samples efficiency. All the obtained extracts were evaluated using a liquid chromatograph coupled with a mass spectrometer (ESI-QTOF–MS). The use of combined chromatographic and spectroscopic techniques with a modern extraction method, allowed us to optimize PLE and to achieve high recovery of alkaloids. This made it possible to compare their content in individual solvents of different polarity. PLE proved to be quick method of comparing and selecting the best solvents for the extraction and isolation of AChE inhibitors with the highest efficiency in plant extracts obtained from the two *Lycopodium* species.

## 2. Results and Discussion

### 2.1. Optimization of PLE and SPE Purification

The extracts from dried aerial parts of *L. clavatum* and *L. annotinum* prepared by using PLE with different solvents were compared in terms of total alkaloids content. The aim of this study was to analyze five different solvents and solutions of different polarity as follows: methanol, 1% methanolic tartaric acid, ethyl acetate, dichloromethane and cyclohexane. The plant materials were extracted using PLE in conditions previously outlined and thoroughly tested for other types of the alkaloids in our laboratory [29,31,32,33]. On the basis of these data, the highest recovery of alkaloids was obtained at elevated temperature of 80 °C and 110 bar pressure. Due to the low availability of plant materials and awareness that these are endangered species, we were only able to use these specific temperature parameters, but providing the best extraction results. Extraction was carried out twice using a particular solvent to obtain the greatest accuracy of tests and to ensure that no significant errors occurred during the whole extraction and analysis procedure. The flexibility in setting various parameters and high automation of the extraction process allowed us to shorten the extraction time and compare the used solvents relatively quickly. Compared to other conducted and described extraction methods, PLE has numerous advantages, e.g., lower solvent consumption, process speed, high automation, separation of the matrix from the extract during extraction and the possibility of changing the solvent. More research is needed to better understand the extraction mechanism, to remove technical barriers and to improve the extraction method to obtain a huge quantity of alkaloids. The optimized PLE has proved more selective in comparison with conventional methods of extraction. It gave highly reproducible results, and it could be easily combined with other techniques such as high performance liquid chromatography/electrospray-ionization-time-of-flight-mass spectrometry (HPLC/ESI-QTOF–MS). The extracts obtained in this study were subjected to further processes. The crude extracts were purified by solid-phase extraction (SPE) in the above-mentioned conditions. We used special Oasis HLB sorbent for selective concentration of the active compounds from crude extracts. It has proven its effectiveness for the isolation of other types of the alkaloids studied in our lab [31,32]. Moreover, this method is relatively fast as in short time several extracts were purified. It enabled us to obtain preliminary purification of alkaloids extracts from the excess ballast substance such as chlorophyll and further identification of alkaloids by HPLC/ESI-QTOF–MS.

### 2.2. Influence of Solvents Used in the Extraction Method on the Composition of the Extracts Identified by HPLC/ESI-QTOF–MS

In this study, five different solvents and solutions of different polarities were evaluated at the above-mentioned conditions (Section 3.2). The obtained extracts were purified by SPE (Section 3.3). Quantities of isolated alkaloids from *L. clavatum* and *L. annotinum* were measured using HPLC/ESI-QTOF–MS. On the basis of the peak areas intensities recorded on the base peak chromatograms (BPCs), we calculated the content of the main detected alkaloids. Five main alkaloids were selected for each species, in all extracts in different concentrations in terms of the percentage to the total amount of alkaloids in a given extract based on the analysis of the BPC. On the basis of these data, we calculated the average content of a given alkaloid and then the mean standard deviation. A surprisingly high alkaloid content in the extracts of two *Lycopodium* species was obtained using different solvents. One of them, lycopodine, the most abundant alkaloid commonly found in *Lycopodium clavatum*, also achieved a high yield in our research. What is more, this compound has occurred in each used solvent and it constituted a high percentage of the samples apart from cyclohexane extract. The lycopodine content even exceeded half of the percentage of all isolated alkaloids extract. For methanol and 1% methanolic tartaric acid extracts almost similar results of lycopodine were obtained. However, this compound was better extracted in dichloromethane. This solvent was previously used in the extraction, but the process was very tedious and time consuming and the yield of extracts was less than 0.5% [4]. Table 1 presents a quantitative analysis of the major compounds identified in *L. clavatum* extracts. The highest amounts of alkaloids were obtained for 1% methanolic tartaric acid and then for methanol extracts. However, in the methanol extract all required alkaloids were detected.

In the case of *L. annotinum*, all alkaloids were identified in methanol and 1% methanolic tartaric acid extracts (Table 2). However, in the methanol extract the yield of individual alkaloids was higher. Despite this, the highest values of one of the main alkaloids of this species-annotinine were obtained using dichloromethane and ethyl acetate. Similar results were observed in these extracts for acrifoline. The high concentration of these alkaloids affected the high sum of all isolated alkaloids in each extract. While comparing the amount of dihydrolycopodine in the two analyzed species, it was found that a better source of this alkaloid was *L. clavatum* (Table 1). Its recovery with three extraction solvents exceeded 15%.

Interestingly, this alkaloid in the ethyl acetate extract of *L. clavatum* had insignificant recovery, and for the *L. annotinum* extract it was not detected at all. On the other hand, to isolate annofoline, it would be better to carry out PLE with only ethyl acetate as a solvent (yield was 28.16%). Comparing the percentage of the total content of the analyzed main alkaloids in particular extracts, a great similarity was observed for both species. The exception was a large difference for 1% methanolic tartaric acid (*L. clavatum* = 72% and for *L. annotinum* = 20.04%). This may be due to the different polarity of alkaloids obtained from *L. annotinum*. Nevertheless, the addition of 1% tartaric acid in methanol was effective for *L. clavatum* extracts, as was the extraction of pyrrolizidine alkaloids from roots of comfrey (*Symphytum officinale* L.) [33]. On the other hand, in the case of *L. annotinum*, the total amount of analyzed compounds increased as the polarity of the extraction solvents decreased.

Although, when analyzing the SD coefficient in the tables (Table 1 and Table 2), it is methanol that had the lowest values. This showed the stability of the extraction process using this solvent. Due to the highest polarity of methanol in comparison with other solvents, we assume that it is the most stable solution in this method. Methanol had a higher sensitivity compared to the solvents used. This was proved by SD < 1.5 in methanol extracts. However, methanol was not the best extraction solvent to isolate a particular *Lycopodium* alkaloid. Moreover, no amounts of alkaloids were identified in cyclohexane extracts belonging to the non-polar solvent. The analyzed mass chromatograms showed no significant homogenous peaks with distinct signals of any compounds for cyclohexane extracts.

### 2.3. LC–MS Identification of Isolated Alkaloids

**Figure 1:**
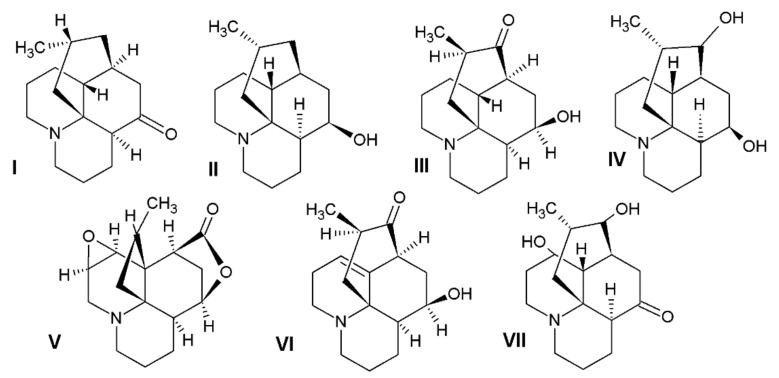
The chemical structures of the alkaloids detected in the investigated plant materials: I—lycopodine, II—dihydrolycopodine, III—annofoline, IV—deacetylfawcettine, V—annotinine, VI—acrifoline, VII—8-β,11-α dihydroxylycopodine.

**Figure 2:**
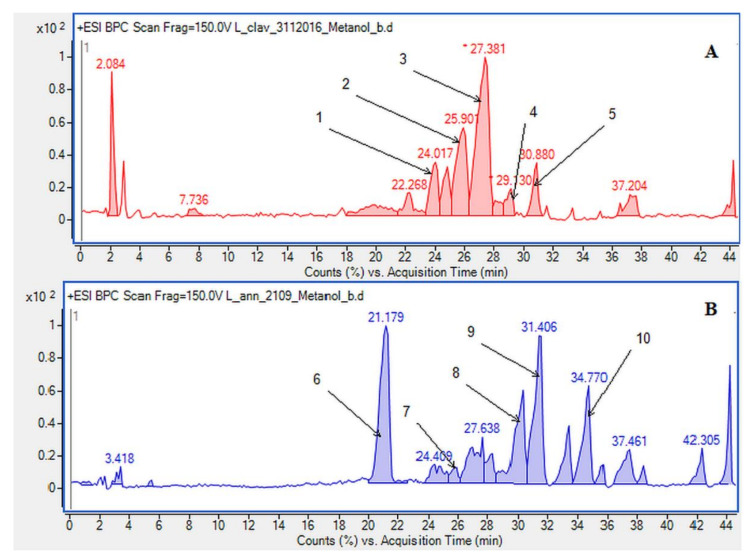
Base peak chromatograms (BPCs) showing the alkaloids identified in the methanolic extracts determined by HPLC/ESI-QTOF–MS in (**A**) *Lycopodium clavatum* L.: 1. annofoline, 2. dihydrolycopodine, 3. lycopodine, 4. deacetylfacettiine and 5. 8-β,11-α dihydroxylycopodine (or epimer). In (**B**) *Lycopodium annotinum* L.: 6. annotinine, 7. dihydrolycopodine, 8. acrifoline, 9. annofoline (or epimer) and 10. 8-β,11-α dihydroxylycopodine (or epimer).

In our research, we obtained alkaloids known in the literature to be common in the *L. clavatum* and *L. annotinum* (Figure 1). To confirm the results and determine the structures of the postulated alkaloids, HPLC/ESI-QTOF–MS analysis was performed. Selected abundant peak results were characteristic of the main *Lycopodium* alkaloids and mentioned in some publications [4,9,17]. The extracts from *L. clavatum* showed abundant peaks areas for lycopodine, dihyrolycopodine, annofoline, deacetylfawcettiine and proposed compound 8-β,11-α dihydroxylycopodine. In case of *L. annotinum* samples we found no significant differences in qualitative composition of extracts, except for high annotinine and acrifoline content (Figure 2). These compounds were subjected to examination using ESI-QTOF–MS in the positive ion mode. As a result, high resolution MS spectra were obtained and components were identified by comparing MS spectra with the literature. The identified alkaloids had the characteristic high resolution mass-to-charge rations for [M + H]+ or M+ ions. The HPLC/ESI-QTOF method has become a useful tool for the confirmation and identification of alkaloids belonging to the Lycopodiaceae family. It is an efficient technique that combines chromatographic separation of plant materials with high resolution mass spectrometry analysis. A careful qualitative analysis of the studied extracts provided the identification of the main alkaloids. The identified compounds are presented below together with the obtained mass chromatograms showing the rich composition of methanol extracts in the established conditions. According to the BPC chromatograms determined by HPLC/ESI-QTOF–MS, peaks were separated, indicating that the various components of *Lycopodium* in methanol extracts could be well separated (Figure 2).

### 2.4. Chemical Composition of Lycopodium clavatum L. and Lycopodium annotinum L.

**Figure 3:**
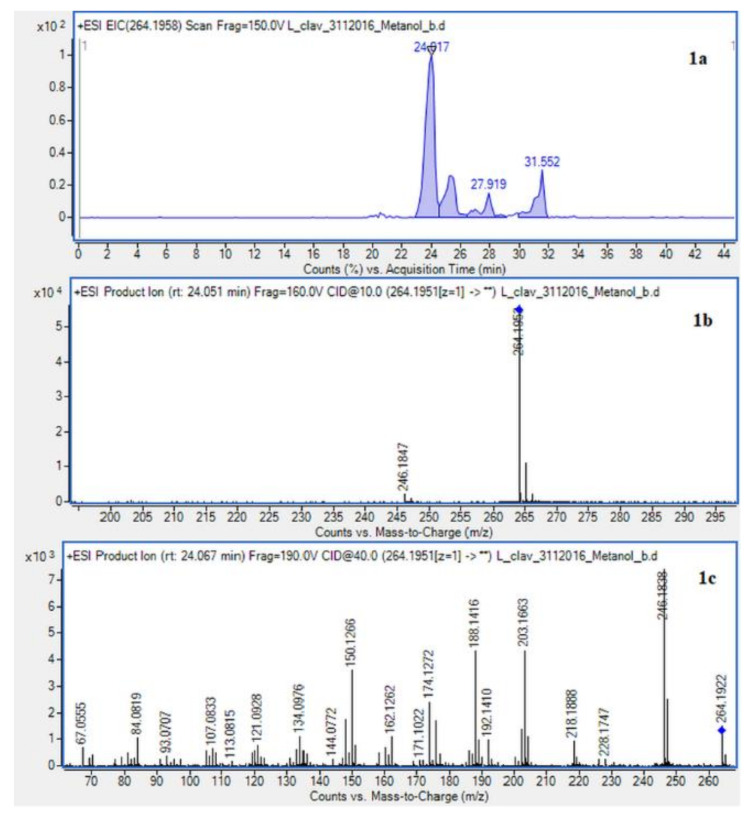

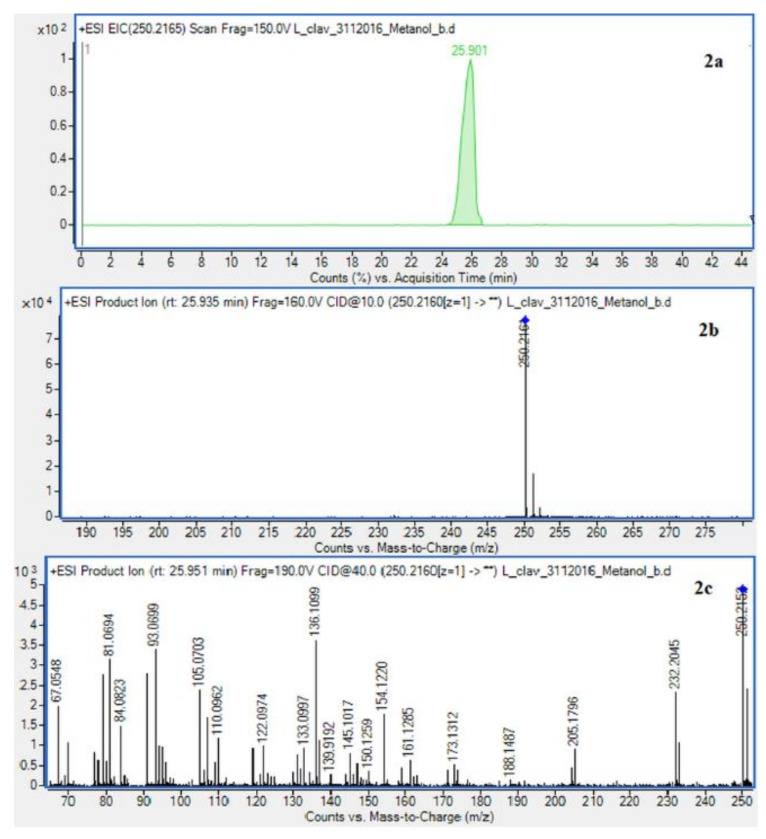

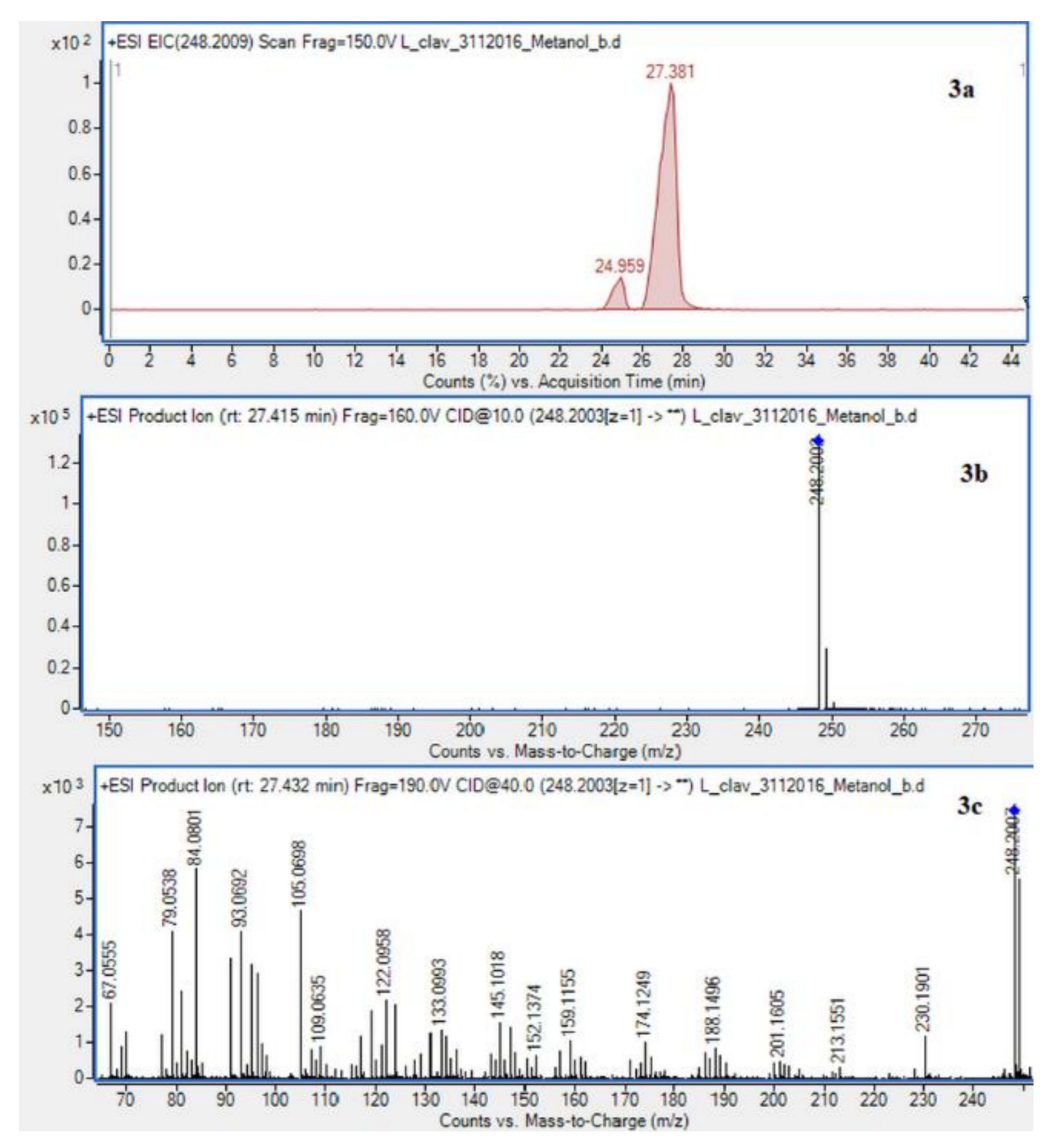

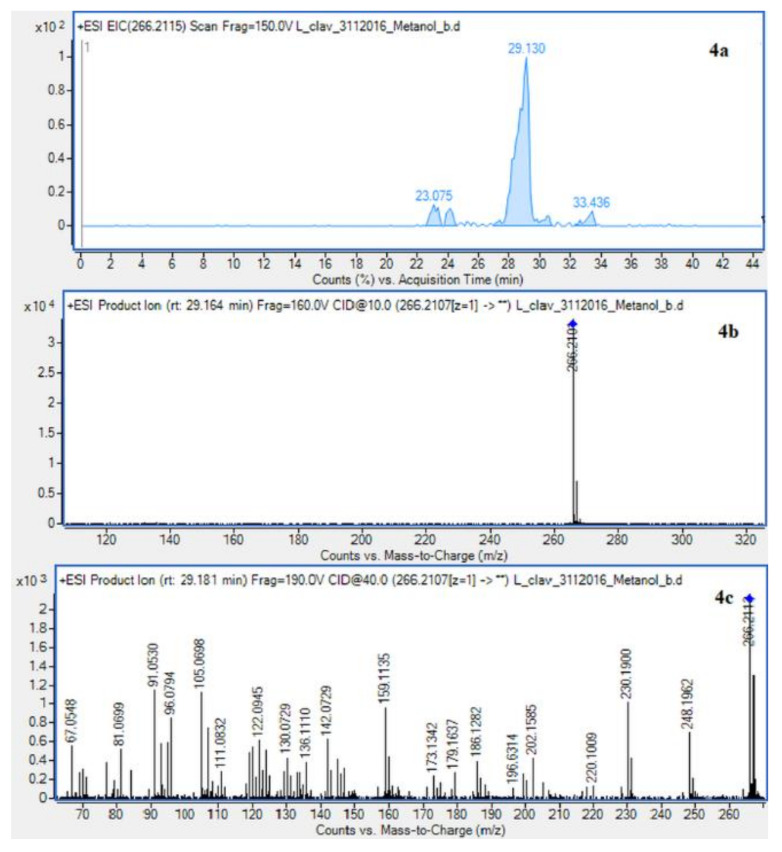

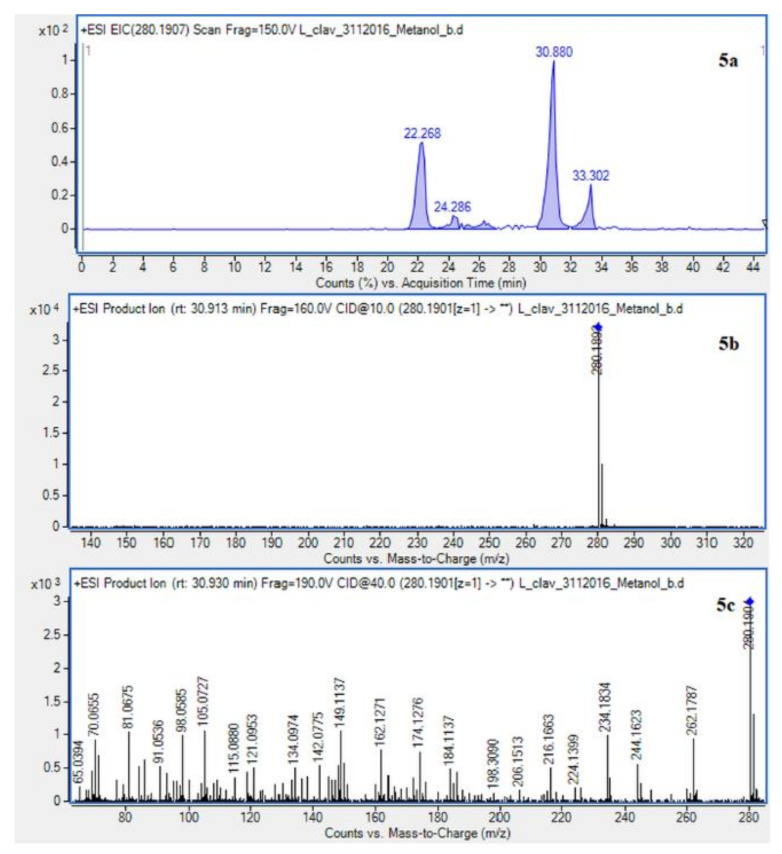
Extracted ion chromatogram (EIC) of *L. clavatum* methanolic extract determined by HPLC/ESI-QTOF–MS showing identified alkaloids: (**1a**)-annofoline at *m*/*z* 264.1958; (**2a**)-dihydrolycopodine at *m*/*z* 250.2165; (**3a**)-lycopodine at *m*/*z* 248.2009; (**4a**) deacetylfawcettiine at *m*/*z* 266.2115 and (**5a**) 8-β,11-α dihydroxylycopodine (or epimer) at *m*/*z* 280.1907. Below CID MS/MS product ion spectra using 10 eV (**b**) and 40 eV (**c**) of collision energy showing the protonated molecules fragmentation pathways.

The presence of *Lycopodium* alkaloids has been confirmed as a result of conducted experiments (Figure 2). Their determination and identification was based on high mass accuracy collision induced dissociation (CID) spectra and analysis of protonated molecules fragmentation pathways and characteristic fragment ions. In general, mass error was below 1 ppm. The *Lycopodium clavatum* L. species is well known for its rich alkaloids content such as lycopodine, dihydrolycopodine and annofoline. One of them, lycopodine, the most abundant alkaloid commonly isolated from *Lycopodium clavatum*, is presented (Figure 3). For the abundant peak at Rt = 27.381 min of the protonated equal to *m*/*z* 248.2009 assigned to lycopodine with *m*/*z*: 230.1901, 145.1018 and 105.0698 fragments (CID, 40 eV). Another peak at Rt = 25.901 min for protonated equal to *m*/*z* 250.2165 was assigned to dihydrolycopodine, where product ions at *m*/*z*: 232.2045, 145.1017 and 136.1099 supported the existence of this alkaloid (CID, 40 eV). The other alkaloids were identified in the similar way (Figure 3).

Due to the still insufficient knowledge and lack of new data on *Lycopodium* alkaloids, the presence of the abundant peaks at Rt = 30.880 (*L. clavatum*) and Rt = 34.770 min (*L. annotinum*) in the methanolic extracts was not clear (Figure 2). Having read several scientific publications on the *Lycopodium* genus and compared them with the presented results it cannot be clearly stated that 8-β,11-α dihydroxylycopodine was identified. There are other similar alkaloids, such as 8-β,11-α dihydroxylycopodine and its derivatives like 6𝛼,8𝛽-dihydroxylycopodine or 4𝛼,8𝛽-dihydroxylycopodine [9,34]. However, examining the fragment pathway, this compound is tentatively classified into the lycopodine class. Peaks 5 and 10 reported in two *Lycopodium* genus (Figure 2) had the same [M + H]+ ion at *m*/*z* 280.1907 and very similar fragment ions at *m*/*z* 262, 244, 218 and 188. The distinguished fragment at *m*/*z* 218 was observed in the MS/MS spectrum indicating a neutral loss of O. The fragment ion at *m*/*z* 188 was elucidated as the loss of C4H8 (56 Da) from an abundant fragment at *m*/*z* 244, characteristic for the lycopodine class. Moreover, the difference in retention time for these two peaks 5 and 10 obtained from the two *Lycopodium* species may indicate that these are tentatively or epimers. The same is evident in the tentative analysis of annofoline (or epimer), which has been identified in both *Lycopodium* species. This alkaloid is present only in methanol and 1% methanolic tartaric acid extracts of the two analyzed *Lycopodium* species at different retention times. Based on the literature and the found molecular ion accurate peak at *m*/*z* 264.1958 and extracted ion chromatogram (EIC) chromatogram, the presence of the two different isomers with retention times is suggested. As shown by the example of methanol extracts: *L. clavatum*-Rt = 24.017 min and *L. annotinum*-Rt = 31.406 min (Figure 2).

**Figure 4:**
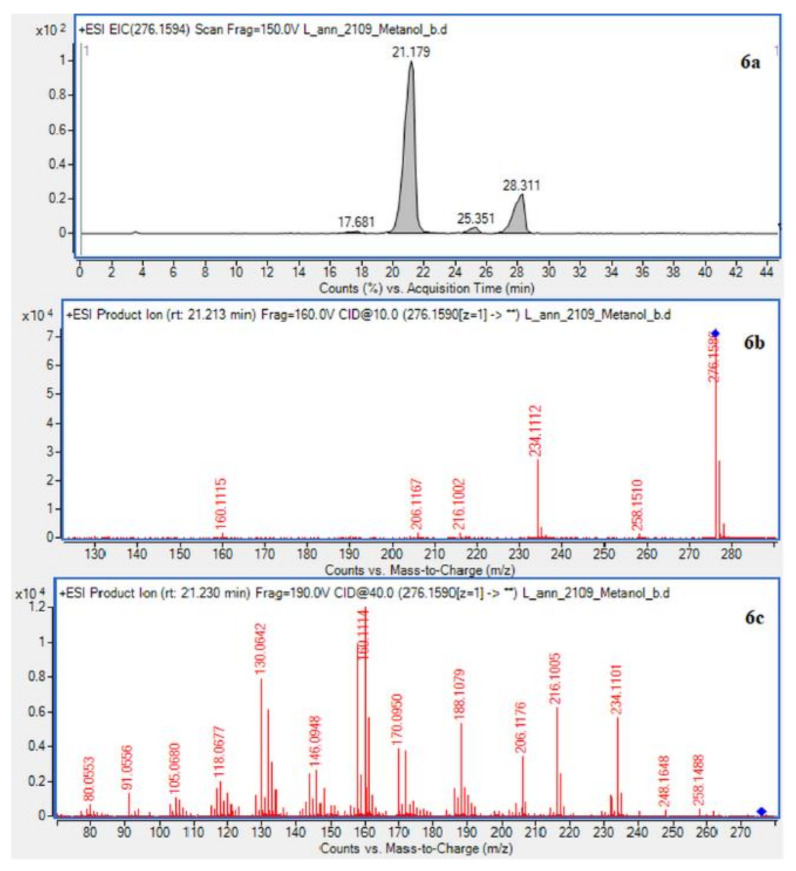

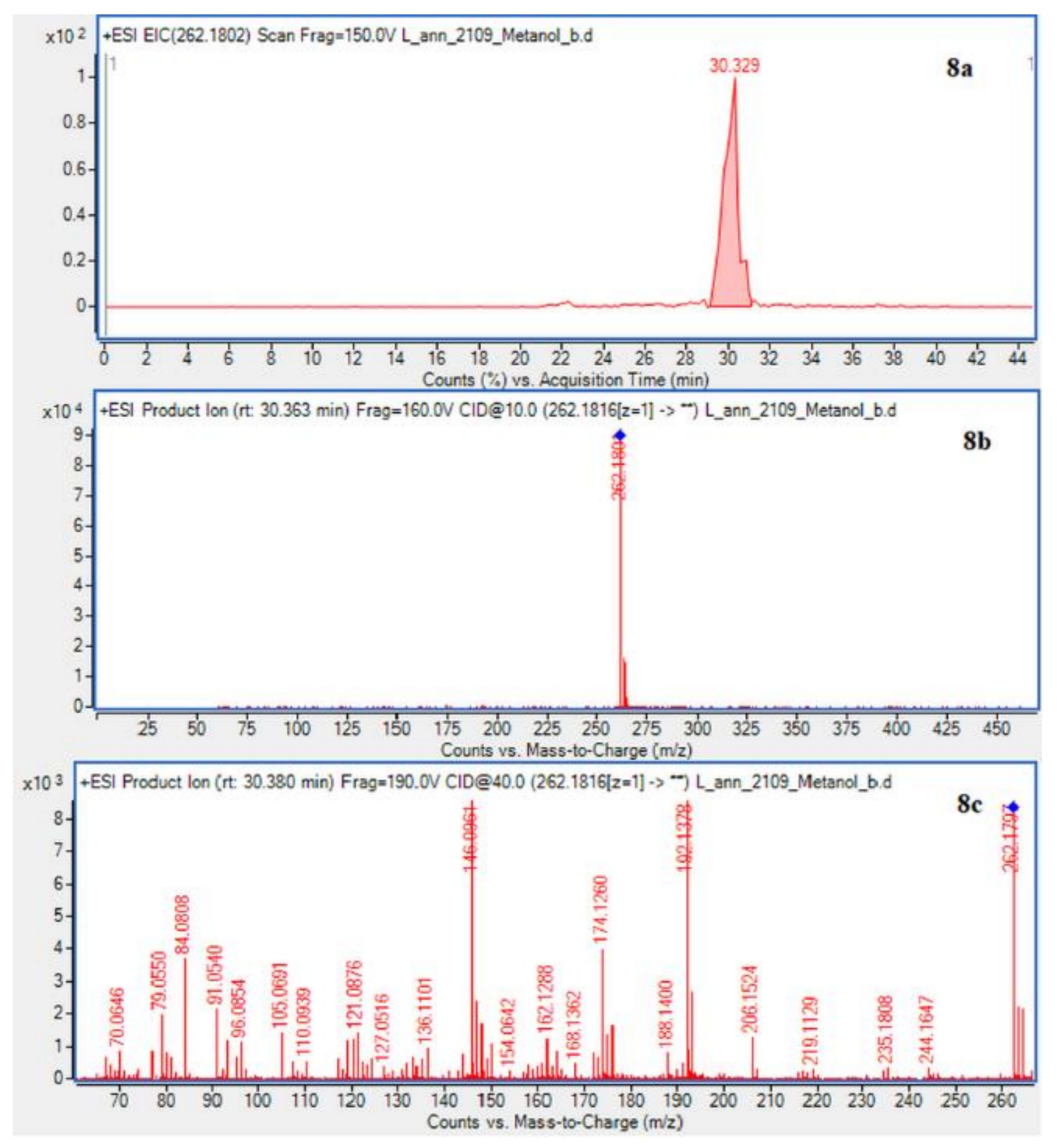
EIC of *L. annotinum* methanol extract determined by HPLC/ESI-QTOF–MS showing alkaloids: (**6a**)-annotinine at *m*/*z* 276.1594 and (**8a**)-acrifoline at *m*/*z* at 262.1802. Below CID MS/MS product ion spectra using 10 eV (**b**) and 40 eV (**c**) of collision energy showing the protonated molecules fragmentation pathways.

In case of *L. annotinum* extracts, high contents of annotinine and acrifoline were detected (Figure 4). The alkaloid with a molecular ion peak [M + H]+ at *m*/*z* 276.1594 (Rt = 21.179 min) identified with characteristic fragments at 234.1101 and *m*/*z* 160.1114 was observed, typical for annotinine. The alkaloid showing the apparent molecular peak [M + H]+ with 262.1816 (Rt = 30.329) for the *L. annotinum* methanolic extract was recognized as acrifoline. Mass spectrum and fragmentation pathway of annotinine and acrifoline were shown in Figure 4. All alkaloid compounds isolated from individual extracts have been identified in the same way for each *Lycopodium* species.

## 3. Materials and Methods

### 3.1. Plant Materials

The plant materials used for research were two species of *Lycopodium*: *Lycopodium clavatum* L. and *Lycopodium annotinum* L. belonging to the Lycopodiaceae family. The plant specimens were collected in Western Ukraine (Rivne Oblast, Sarny region) and received and authenticated by the authors of this study. Only the aerial parts of plant materials were used for the research in the amount of 100 g for each species and then dried in an oven at 28 °C. Previously, the shoots were grounded in an electric grinder and then sieved through a sieve with a mesh diameter of 0.5 mm, in accordance with the requirements of the Polish Pharmacopoeia. The samples of these plants are deposited in the Independent Laboratory of Chemistry of Natural Products, Medical University of Lublin in Poland.

### 3.2. Samples Preparation and Extraction Conditions

For each experiment, 1.0 g of grounded powder from the dried plant materials was placed in a 10 mL stainless steel extraction cell. Extraction of alkaloids was performed by PLE at an elevated temperature of 80 °C and 110 bar pressure using a range of solvents for comparison: methanol, ethyl acetate, 1% tartaric acid solution in methanol, dichloromethane and cyclohexane. The working conditions of the Dionex ASE 100 extractor were set as follows: static time 10 min, flush volume 60%, number of extraction cycles 3; for each solvent, the tests were performed twice. This selection of parameters was the result of a number of previous studies conducted in our laboratory [29,31,32,33]. The obtained extracts were transferred into 50 mL volumetric flasks and diluted to the mark with the solvent used for extraction. These extracts were further purified by SPE.

### 3.3. Purification of Extracts by the Solid-Phase Extraction

From each flask, 20 mL of the obtained extracts was evaporated on a rotary vacuum evaporator at 50 °C, and then was purified using SPE. Dry residues were dissolved in 3 mL of the mixture water–methanol (90:10, *v/v*) with adding 2 drops of 10% ammonia, and applied onto previously conditioned cartridge. In this technique, a cartridge (Oasis HLB 3cc) was at first conditioned with 3 mL of methanol, then with 3 mL of water–methanol (90:10, *v/v*) with 2 drops of 25% ammonia using vacuum and a flow rate of 1 drop/1 s. About a 1 mm thin layer of the solvent deliberately was remained. Fractions of alkaloids were subsequently flushed out with 8 mL of methanol–water (75:25, *v/v*) mixture containing 10 mM of ammonium formate at pH 3.5 regulated with 0.8 mL 10% of formic acid, and diluted to 10 mL with methanol for further analyses. The purified extracts were analyzed using HPLC/ESI-QTOF–MS.

### 3.4. LC–MS Identification of the Main Isolated Alkaloids

HPLC/ESI-QTOF–MS analysis were performed using an Agilent Technologies 6530 B system in the positive ion mode with an ESI-Jet Stream^®^ ion source. The liquid chromatograph was equipped with 6530 B mass spectrometer with a quadrupole time-of-flight mass (QTOF) analyzer, gradient pump, diode array detector (DAD), autosampler and column oven. The analytical silica column was a 3.5 µm, 150 mm × 2.1 mm Atlantis HILIC (Waters, Milford, MA, USA). The mobile phase consisted of solvent A: acetonitrile (95%) with 10 mM ammonium formate (0.2%) and solvent B: acetonitrile (50%) with 10 mM ammonium formate (0.2%). The following gradient procedure was adopted: 0–10 min, 100% using solvent A; 10–40 min, 92% solvent A and 8% solvent B; 40–45 min, 64% solvent A and 36% of solvent B. Total analysis time was 45 min with a stable flow rate at 0.25 mL/min. The injection volume was 1 µL of extracts. Analyses were performed according to the following parameters of the dual spray jet stream ESI ion source in positive ion mode: fragmentor voltage—120 V, nitrogen flow—10 L/min, gas temperature—350 °C, sheath gas temperature—325 °C and sheath gas—10 L/min, nebulizer pressure was set at level of 35 psi. The range of measured *m*/*z* was 100–1000 units in the Auto MS/MS acquisition mode. Skimmer voltage-65 V and octopole RF Peak-750 V. CID was conducted at two different energies 10 and 40 eV with the MS scan rate 1 spectrum per s, 2 spectra per cycle. Mass Hunter 2.2.1 (Agilent Technologies, Santa Clara, CA, USA) running under a Windows system was used for the data acquisition and analysis.

## 4. Conclusions

For the first time, a novel and efficient method of *Lycopodium* alkaloids extraction has been developed. PLE was optimized using different solvents to compare, so as to select the best conditions for the alkaloids extraction of the two species: *Lycopodium clavatum* L. and *Lycopodium annotinum* L. Modern PLE in combination with SPE allowed us to obtain results with high accuracy. Moreover, it enabled us to select the best solvent for the isolation of the four groups of *Lycopodium* alkaloids. To the best of our knowledge, this is the first report of a comparison of *Lycopodium* alkaloids content obtained from the two species collected in Ukraine, suggesting that the kind of solvents exerted a great influence on the alkaloid isolation efficiency. Among the results obtained, lycopodine achieved high yields in dichloromethane and 1% methanolic tartaric acid extracts. For the first time, this compound exceeded the yield of more than 30%. In the case of *L. annotinum*, the highest value was achieved for annotinine (over 40%) in ethyl acetate and dichloromethane extracts. Such alkaloid recoveries have not been obtained so far. Additionally, higher dihydrolycopodine recoveries were observed in *L. clavatum*, especially in the 1% methanolic tartaric acid extract. On the other hand, when comparing the total percentage recovery of the analyzed alkaloids in both *Lycopodium* species, for *L. clavatum* the highest values were obtained in 1% methanolic tartaric acid extract (in contrast to *L. annotinum* extract), and for *L. annotinum* in dichloromethane extract. No amounts of alkaloids were detected in cyclohexane with non-polar solvent. In turn, high polarity of methanol in comparison with other solvents, confirmed the highest stability of the obtained results (SD < 1). What is more, due to the use of methanol as a solvent for extraction, we obtained chromatograms with all isolated alkaloids and the best separation and quality of the bands. Based on these results, it could be concluded that PLE is a preferable technique for the recovery of *Lycopodium* alkaloids. These novel techniques proved to be an efficient and reliable method for the quantitative recovery of alkaloids. In this novel study, we demonstrated that a selection of solvents or solutions with differing molecular mass and polarity resulted in various yields of obtained alkaloids. Besides, an attempt to obtain high efficiency of the process has given unprecedented effectiveness of the solvents used. The initiated techniques may become the only method to isolate individual *Lycopodium* alkaloids in the future.

## Figures and Tables

**Table 1:** Pressurized liquid extraction (PLE) of *Lycopodium clavatum* L. using five kind of solvent systems (Section 3.2) and purified by solid-phase extraction (SPE) procedure (Section 3.3) and the five main alkaloids were isolated with different efficiency.

Proposed Compound	Annofoline		Dihydrolycopodine		Lycopodine		Deacetylfawcettiine		8-β,11-α Dihydroxylycopodine		% SUM of Isolated Alkaloids
Extraction Solvent	avg. (%)	±SD	avg. (%)	±SD	avg. (%)	±SD	avg. (%)	± SD	avg. (%)	±SD	
Methanol	7.34	0.35	16.27	1.20	33.85	0.51	4.76	1.07	5.91	0.94	68.13
1% methanolic tartaric acid	9.90	0.47	21.46	2.14	38.13	3.24	n.d.t	0	2.51	1.17	72.0
Ethyl acetate	28.16	3.09	1.78	0.11	17.86	2.62	n.d.t	0	n.d.t	0	47.8
Dichloromethane	3.99	0.49	15.87	3.27	45.82	2.52	n.d.t	0	n.d.t	0	65.68
Cyclohexane	n.d.t	0	n.d.t	0	n.d.t	0	n.d.t	0	n.d.t	0	0

avg. (%)—the average concentration (%) of isolated compounds to the total amount of all alkaloid compounds obtained from the two extracts based on analysis of the total ion current (TIC)chromatograms.

**Table 2:** PLE of *Lycopodium annotinum* L. using five kind of solvents systems (Section 3.2) and purified by SPE procedure (Section 3.3). The obtained extracts were identified by HPLC/ESI-QTOF (Section 3.4) and the five main alkaloids were isolated with different efficiency.

Proposed Compound	Annotinine		Dihydrolycopodine		Acifoline		Annofoline or Epimer		8-β,11-α Dihydroxylycopodine or Epimer		% SUM of Isolated Alkaloids
Extraction Solvent	avg. (%)	±SD	avg. (%)	±SD	avg. (%)	±SD	avg. (%)	±SD	avg. (%)	±SD	
Methanol	21.11	0.79	1.93	0.34	12.59	0.70	17.17	0.30	9.83	0.34	62.63
1% methanolic tartaric acid	9.45	1.34	1.52	0.68	2.53	2.32	3.92	3.76	2.98	1.34	20.04
Ethyl acetate	41.79	3.85	n.d.t	0	20.66	1.53	2.38	0.90	n.d.t	0	64.83
Dichloromethane	44.12	2.04	1.27	0.33	22.41	1.61	10.17	3.87	n.d.t	0	77.97
Cyclohexane	n.d.t	0	n.d.t	0	n.d.t	0	n.d.t	0	n.d.t	0	0

avg. (%)—the average concentration (%) of isolated compounds to the total amount of all alkaloid compounds obtained from the two extracts based on analysis of the TIC chromatograms.

## Data Availability

The data confirming the results obtained are deposited in the Independent Laboratory of Chemistry of Natural Products, Medical University of Lublin. They are available from the corresponding author upon reasonable request.

## References

[1] Da Silva G.F., Gandolfi P.H.K., Almeida R.N., Lucas A.M., Cassel E., Vargas R.M.F. (2015). Analysis of supercritical fluid extraction of lycopodine using response surface methodology and process mathematical modeling. Chem. Eng. Res. Des..

[2] Banerjee J., Biswas S., Madhu N.R., Karmakar S.R., Biswas S.J. (2014). A better understanding of pharmacological activities and uses of phytochemicals of *Lycopodium clavatum*: A review. J. Pharmacogn. Phytochem..

[3] Zangara A. (2003). The psychopharmacology of huperzine A: An alkaloid with cognitive enhancing and neuroprotective properties of interest in the treatment of Alzheimer’s disease. Pharmacol. Biochem. Behav..

[4] Konrath E.L., Neves B.M., Lunardi P.S., Passos C.S., Simoes-Pires A., Ortega M.G., Goncalves C.A., Cabrera J.L., Moreira J.C.F., Henriques A.T. (2012). Investigation of the in vitro and ex vivo acetylcholinesterase and antioxidant activities of traditionally used *Lycopodium* species from South America on alkaloid extracts. J. Ethnopharmacol..

[5] Orhan I., Kupeli E., Sener B., Yesilada E. (2007). Appraisal of anti-inflammatory potential of the clubmoss, *Lycopodium clavatum* L.. J. Ethnopharmacol..

[6] Mandal S.K., Biswas R., Bhattacharyya S.S., Paul S., Dutta S., Pathak S., Khuda-Bukhsh A.R. (2010). Lycopodine from *Lycopodium clavatum* extract inhibits proliferation of HeLa cells through induction of apoptosis via caspase-3 activation. Eur. J. Pharmacol..

[7] Pathak S., Kumar Das J., Jyoti Biswas S., Khuda-Bukhsh A.R. (2006). Protective potentials of a potentized homeopathic drug, Lycopodium-30, in ameliorating azo dye induced hepatocarcinogenesis in mice. Mol. Cell. Biochem..

[8] Pathak S., Banerjee A., Paul S., Khuda-Bukhsh A.R. (2009). Protective potentials of a plant extract (*Lycopodium clavatum*) on mice chronically fed hepato-carcinogens. Indian. J. Exp. Biol..

[9] Li X., Kang M., Ma N., Pang T., Zhang Y., Jin H., Yang Z., Song L. (2019). Identification and analysis of chemical constituents and rat serum metabolites in *Lycopodium clavatum* using UPLC-Q-TOF/MS combined with multiple data-processing approaches. Evid. Based Complement Alternat. Med..

[10] Cummings J.L., Vinters H.V., Cole G.M., Khachaturian Z.S. (1998). Alzheimer’s disease: Etiologies, pathophysiology, cognitive reserve, and treatment opportunities. Neurology.

[11] Ferreira A., Rodrigues M., Fortuna A., Falcao A., Alves G. (2016). Huperzine A from *Huperzia serrata*: A review of its sources, chemistry, pharmacology and toxicology. Phytochem. Rev..

[12] Wang R., Yan H., Tang X.-C. (2006). Progress in studies of huperzine A, a natural cholinesterase inhibitor from Chinese herbal medicine. Acta. Pharmacol. Sin..

[13] Wang L.M., Han Y.F., Tang X.-C. (2000). Huperzine A improves cognitive deficits caused by chronic cerebral hypoperfusion in rats. Eur. J. Pharmacol..

[14] Pepping J. (2000). Huperzine A. Am. J. Health. Syst. Pharm..

[15] Orhan I., Terzioglu S., Sener B. (2003). Aplha-onocerin: An acetylcholinesterase inhibitor from *Lycopodium clavatum*. Planta Med..

[16] Zuo Z.-X., Wang Y.-J., Liu L., Wang Y., Mei S.-H., Feng Z.-H., Wang M., Li X.-Y. (2015). Huperzine A alleviates mechanical allodynia but not spontaneous pain via muscarinic acetylcholine receptors in mice. Neural. Plast..

[17] Ma X., Gang D.R. (2004). The Lycopodium alkaloids. Nat. Prod. Rep..

[18] Thorroad S., Worawittayanont P., Khunnawutmanotham N., Chimnoi N., Jumruksa A., Ruchirawat S., Thasana N. (2014). Three new *Lycopodium* alkaloids from *Huperzia carinata* and *Huperzia squarrosa*. Tetrahedron.

[19] Weller J., Budson A. (2018). Current understanding of Alzheimer’s disease diagnosis and treatment. F1000Research.

[20] De Luca V., Salim V., Atsumi S.M., Yu F. (2012). Mining the biodiversity of plants: A revolution in the making. Science..

[21] Halldorsdottir E.S., Jaroszewski J.W., Olafsdottir E.S. (2010). Acetylcholinesterase inhibitory activity of lycopodane—Type alkaloids from Icelandic *Lycopodium annotinum* spp. alpestre. Phytochemistry.

[22] Tian Y.-Q., Hu G.-W., Guo M.-Q. (2016). Components and anti-HepG2 activity comparison of *Lycopodium* alkaloids from four geographic origins, Evid. Based Complement. Alternat. Med..

[23] Zhang H., Liang H., Kuang P., Yuan Q., Wang Y. (2012). Simultaneously preparative purification of Huperzine A and Huperzine B from *Huperzia serrata* by macroporous resin and preparative high performance liquid chromatography. J. Chromatogr. B.

[24] Kim J., Choi Y.M., Yoo K.-P., Pelletetier S.W. (2001). Supercritical fluid extraction of alkaloids. Alkaloids: Chemical and Biological Perspectives.

[25] Kohler M., Haerdi W., Christen P., Veuthey J.-L. (1997). Extraction of artemisinin and artemisinic acid from *Artemisia annua* L. using supercritical carbon dioxide. J. Chromatogr. A.

[26] Wang L., Weller C.L. (2006). Recent advances in extraction of nutraceuticals from plants. Trends Food Sci. Tech..

[27] Ahmad R., Ahmad N., Al-Anaki W.S., Ismail F.A., Al-Jishi F. (2020). Solvent and temperature effect of accelerated solvent extraction (ASE) coupled with ultra-high-pressure liquid chromatography (UHPLC-PDA) for the determination of methyl xanthines in commercial tea and coffee. Food Chem..

[28] Czernicka L., Ludwiczuk A., Rój E., Marzec Z., Jarzab A., Kukula-Koch W. (2020). Acetylcholinesterase inhibitors among *Zingiber officinale* terpens—Extraction conditions and thin layer chromatography- based bioautography studies. Molecules.

[29] Mroczek T., Mazurek J. (2009). Pressurized liquid extraction and anticholinesterase activity-based thin-layer chromatography with bioautography of *Amaryllidaceae* alkaloids. Anal. Chim. Acta..

[30] Souza M.C., Silva L.C., Chaves J.O., Salvador M.P., Sanches V.L., da Cunha D.T., Carneiro T.F., Rostagno M.A. (2021). Simultaneous extraction and separation of compounds from mate (*Ilex paraguariensis*) leaves by pressurized liquid extraction coupled with solid-phase extraction and in-line UV detection. Food Chem. Mol. Sci..

[31] Mroczek T., Dymek A., Widelski J., Wojtanowski K.K. (2020). The bioassay-guided fractionation and identification of potent acetylcholinesterase inhibitors form *Narcissus c.v. ‘Hawera’* using optimized vacuum liquid chromatography, high resolution mass spectrometry and bioautography. Metabolites.

[32] Mroczek T. (2016). Qualitative and quantitative two-dimensional thin-layer chromatography/high performance liquid chromatography/diode-array/electrospray-ionization-time-of-flight mass spectrometry of cholinesterase inhibitors. J. Pharm. Biomed. Anal..

[33] Mroczek T., Widelski J., Głowniak K. (2006). Optimization of extraction of pyrrolizidine alkaloids from plant material. Chem. Anal..

[34] Pongpamorn P., Wan-erlor S., Ruchirawat S., Thasana N. (2016). Lycoclavatumide and 8β,11α-dihydroxylycopodine, a new fawcettimine and lycopodine-type alkaloid from *Lycopodium clavatum*. Tetrahedron.

